# Extracellular vesicles derived from immortalized human natural killer cell line NK3.3 as a novel therapeutic for multiple myeloma

**DOI:** 10.3389/fimmu.2023.1265101

**Published:** 2023-09-25

**Authors:** Emily C. Matchett, Jacki Kornbluth

**Affiliations:** ^1^ Department of Pathology, Saint Louis University School of Medicine, St. Louis, MO, United States; ^2^ Research and Education Service, VA St. Louis Health Care System, St. Louis, MO, United States

**Keywords:** natural killer cells, extracellular vesicles, multiple myeloma, drug resistance, cytotoxicity

## Abstract

**Introduction:**

Over the last decade, there have been many advancements in the therapeutic treatment of multiple myeloma (MM), including the use of natural killer (NK) cells. However, despite promising results from clinical trials, there are concerns over the use of NK cell-based therapy. Cells often undergo growth arrest, limiting their experimental utility; donor cells are extremely heterogeneous, resulting in content variability; and patients receiving allogeneic cells are at risk for graft-versus-host disease and/or cytokine release syndrome. Extracellular vesicles (EVs) have emerged as a new natural therapeutic tool. EVs are known to carry cargo derived from the parent cell from which they originate. NK cells play an important role in the innate immune system, targeting and killing tumor cells. This has led many researchers to isolate EVs from NK cells for their cytotoxic potential.

**Methods:**

In this study, we isolated EVs from the NK cell line, NK3.3, which was derived from the peripheral blood of a healthy donor. Currently, it is the only normal human NK cell line reported with all the functional characteristics of healthy NK cells. To address the issue of growth arrest, we immortalized NK3.3 cells with lentivirus encoding the catalytic subunit of human telomerase *h*tert (NK3.3-LTV). EVs from these cells were isolated using a modified polyethylene glycol (PEG)-acetate precipitation protocol to simplify processing and increase EV yield.

**Results and conclusions:**

We demonstrated that NK3.3-LTV EVs target both sensitive and drug-resistant MM cell lines as well as primary patient MM cells *in vitro*, decreasing proliferation and inducing apoptotic cell death as well as or better than EVs from non-immortalized cells with no toxicity towards normal cells. This study is the first step towards developing an immunotherapeutic product designed to treat patients with relapsed/refractory MM.

## 1 Introduction

Multiple myeloma (MM) is the third most common hematological malignancy and is characterized by the proliferation of abnormal monoclonal plasma cells and increased production of monoclonal immunoglobulin or light chains in the blood and/or urine. Hallmarks of the disease include anemia, renal failure, and osteolytic bone lesions (1, 2).

Many therapeutic advancements have been made to treat MM, including the development of novel agents to target tumor burden and bone disease. Current standard therapy involves autologous stem cell transplantation, followed by high-dose chemotherapy often combined with immunomodulatory drugs (thalidomide, lenalidomide, and pomalidomide) or proteasome inhibitors (bortezomib and carfilzomib) (2). New drug therapy has increased the overall median survival from 2-3 years to 8-10 years. However, despite these advancements, MM patients often acquire multi-drug resistance leading to relapse/refractory MM (3–5).

A promising therapeutic approach is using natural killer (NK) cells. NK cells are an essential component of the innate immune system, whose main function is to destroy virally infected or cancerous cells (6). NK cells rapidly recognize and kill tumor cells without prior sensitization through their ability to distinguish “self” from “non-self” cells. NK cell recognition and activation are controlled by multiple NK receptors. Induction of cytotoxicity is the result of NK receptor engagement of “non-self” stress proteins on abnormal cells and the lack of an inhibitory “self” signal mediated by major histocompatibility complex class I (MHC-1) molecules. MHC-1 is often down-regulated on tumor cells, triggering NK cell activation (7). When an NK cell encounters a target (tumor) cell, cytolytic granules containing perforin and granzymes are released and enter the target cell, inducing apoptosis. NK cells can also induce tumor killing through the secretion of cytokines (IFN-γ, TNF-α) and/or chemokines (chemokine C-C motif ligand 3 (CCL3), CCL4 and CCL5) (7, 8).

Besides releasing cytokines and chemokines into the extracellular space, NK cells also release extracellular vesicles (EVs) (8). EVs are comprised of nanometer-sized microvesicles and exosomes. Microvesicles form from the outward budding of the plasma membrane and measure 100 – 1,000 nm in diameter. Exosomes form from the endosomal compartment of cells and range in size from 30 – 200 nm in diameter (9). They carry a wide variety of cargo, including DNA, messenger RNAs, long noncoding RNAs, microRNAs, lipids, and proteins reflective of the parent cell from which they originate (7, 10). We found that NK3.3 EVs are primarily in the exosome size range, express both NK and EV markers, along with killer proteins, perforin and granzymes, and induce apoptosis of tumor cells (9, 10).

There has been a growing interest in the therapeutic use of NK-derived EVs to treat cancer. Several studies have reported the anti-tumoral effect of NK EVs *in vitro* and *in vivo* most prominently against breast cancer (11–16), glioblastoma (11, 12, 16) and leukemia (15, 17, 18). Until now, there have been no studies testing the anti-tumor activity of NK-derived EVs against MM. Even though NK EVs have demonstrated potent cytotoxicity against tumor cells, they do not affect healthy cells (10, 17).

Compared to cell-based therapies, EVs are considered a safer treatment option due to their inability to proliferate and elicit an immune response. They are easier and less costly to manufacture and can be used as a “natural” drug delivery vehicle (19). One study pre-loaded NK EVs with paclitaxel, a chemotherapeutic, to enhance anti-tumor activity against breast cancer. The pre-loaded NK EVs significantly decreased cell viability and increased apoptosis compared to free paclitaxel (13). Another study used NK EVs and a siRNA-based gene silencing system to target BCL-2 (siBCL-2) in breast cancer. BCL-2 is an anti-apoptotic protein overexpressed in cancer cells. The NK EVs loaded with siBCL-2 demonstrated increased killing activity against breast cancer (15).

Most of these studies employ either primary NK cells or NK92 cells as an EV source. NK92 cells are a continuously growing NK cell line derived from the mononuclear blood cells of a patient with aggressive NK-cell lymphoma (20). Primary NK cells isolated from cancer patients are often dysfunctional due to circulating serum suppression factors or following chemotherapy (20). Allogeneic NK cells often exhibit donor-dependent functional heterogeneity and have a finite lifespan. This can lead to variability across studies and reduce reproducibility (19). NK cell isolation requires donors to be connected to a leukapheresis device for several hours to collect their white blood cells, of which only about 10% are NK cells (20). Furthermore, allogeneic NK cells must be treated to remove T lymphocytes to prevent the risk of a life-threatening graft-versus-host reaction (20). Once isolated, primary NK cells must be activated and expanded to acquire enough cytotoxic NK cells, often with the use of a genetically modified feeder cell line or cytokine stimulation (11, 16, 18, 21, 22).

A novel approach towards NK EV therapy would be to not only have a continuously growing non-tumorigenic NK cell line, but an NK cell line that can produce large quantities of homogeneous, highly cytotoxic EVs. NK3.3 is a one-of-a-kind NK cell line that was generated from the peripheral blood of a healthy donor. We previously described the anti-tumor efficacy of NK3.3 EVs against leukemia and breast cancer (10). In this study, we immortalized NK3.3 cells with a lentivirus encoding the catalytic subunit of human telomerase (*h*tert) to overcome cell exhaustion and promote continuous cell expansion. We isolated EVs from immortalized cells (NK3.3-LTV EVs) by high-speed centrifugation and sterile filtration, followed by a modified polyethylene-glycol (PEG)-acetate precipitation protocol to increase EV yield and purity. We show, for the first time, that NK3.3-LTV EVs inhibit tumor proliferation and induce cell death of several MM cell lines, including primary patient cells and drug-resistant MM cells, but they do not harm normal cells. EVs derived from immortalized NK3.3 cells display similar protein content and cytotoxic activity as EVs from non-immortalized cells. This indicates that NK3.3-LTV EVs are a safe, naturally derived therapeutic for the targeted destruction of MM, especially for treating patients who failed other treatment modalities.

## 2 Materials and methods

### 2.1 Cell lines

The generation of human NK cell line, NK3.3, has been described in detail previously (23). NK3.3 was maintained in RPMI-1640 medium, supplemented with 20% fetal bovine serum (FBS), 1% L-glutamine (L-glut), 1% penicillin-streptomycin (P/S), and 125 U/mL recombinant human IL-2 (Bio-Techne, Minneapolis, MN). The human erythroleukemia cell line, K562, human MM cell line, RPMI-8226, human MM cell line, U266B1 (U266), and human embryonic kidney cell line, HEK293, were purchased from the American Type Culture Collection. MM cell lines ARD, ARP, and MER, derived from bone marrow aspirates of MM patients, were produced in collaboration with Dr. Joshua Epstein at the University of Arkansas Myeloma Center (**Table 1**). All cell lines have MM surface markers, secrete immunoglobulin or light chains, and are Epstein-Barr virus-negative (24–27). The luciferase-transfected MM cell line, RPMI-8226 (8226/Luc) was provided by Dr. Patrick Frost, UCLA (28). The melphalan-resistant MM cell line 8226/LR5 and bortezomib-resistant line, 8226/B25, were gifted by Dr. Kenneth Shain of the Moffitt Cancer Center (29, 30). Human foreskin fibroblasts (hFF) were provided by Dr. Ratna Ray of Saint Louis University.

**Table 1:** Multiple myeloma cell lines.

Cell Line	Ig Isotype	Source
RPMI-8226	λ light chain	ATCC
8226/Luc	λ light chain	Dr. Patrick Frost- UCLA
8226/B25	λ light chain	Dr. Kenneth Shain- MCC
8226/LR5	λ light chain	Dr. Kenneth Shain- MCC
U266	IgE-k	ATCC
ARD	IgA-k	ACRC
ARP	IgA-k	ACRC
MER	IgM-k	ACRC

ACRC, Arkansas Cancer Research Center.

ATCC, American Type Culture Collection.

MCC, Moffitt Cancer Center.

UCLA, University of California, Los Angeles.

ARD, ARP, MER, RPMI-8226, 8226/Luc, 8226/LR5, 8226/B25, U266, and K562 were cultured in RPMI-1640 medium, supplemented with 10% FBS, 1% L-glut, and 1% P/S. Melphalan (5 µM) was added to 8226/LR5 culture media and 8226/B25 media contained 25 nM bortezomib. HEK293 and hFF were cultured in DMEM high glucose medium, supplemented with 10% FBS, 1% L-glut, and 1% P/S. All cells were maintained in an atmosphere of 5% CO_2_ at 37°C with 5% relative humidity. Cell counts were determined using a hemocytometer and cell viability determined using trypan blue dye exclusion (MilliporeSigma, Burlington, MA). All cell lines were tested for mycoplasma using Invivogen Mycoplasma Detection Kit (Thermo Fisher Scientific, Waltham, MA).

### 2.2 Primary MM cells

We obtained bone marrow samples from MM patients during scheduled clinical visits at the University of Arkansas Cancer Center. All patients signed Institutional Review Board-approved informed consent forms. Patient information was de-identified, except for the parameters shown in **Table 2**. Mononuclear cells from heparinized bone marrow aspirates were separated by Ficoll-Hypaque density gradient centrifugation and CD38^+^ MM cells collected by sorting.

**Table 2:** Characteristics of primary myeloma cells derived from the bone marrow of MM patients.

Patient	Sex	Age	Ig Isotype
1	F	60 and older	IgG-κ
2	M	35 – 39 years	IgG-κ
3	M	55 – 59 years	IgG-κ
4	F	55 – 59 years	IgG-κ
5	M	45 – 49 years	IgA-κ
6	M	55 – 59 years	IgA-κ

Bone marrow was collected from MM patients during scheduled clinic visits. All patients signed IRB-approved consent forms. Bone marrow mononuclear cells were obtained by density gradient centrifugation and CD38^+^ tumor cells were isolated by cell sorting.

### 2.3 Generation of immortalized NK3.3 cells

NK3.3 cells were transduced with *h*tert (EF1a, GFP, puro), a replication-incompetent, self-inactivating lentivirus (GenTarget Inc., San Diego, CA) to generate immortalized cells (NK3.3-LTV). Transduced cells were selected for puromycin resistance.

### 2.4 Flow cytometry

NK3.3-LTV cells were analyzed for changes in cell surface marker expression compared to non-immortalized NK3.3 cells. All cells (1 x 10^5^ cells per antibody) were centrifuged at 300 g x 5 min, media aspirated, and 1.5 µL single antibody + 20 µL staining buffer (phosphate buffered saline (PBS) + 1% bovine serum albumin + 1% sodium azide) were added to cells. Sample tubes were incubated for 30 min on ice, followed by addition of 200 µL staining buffer and incubated for another 10 min on ice. Samples were analyzed using LSRFortessa Cell Analyzer (BD Biosciences, Franklin Lakes, NJ). Data were analyzed using FlowJo software. Antibodies used include: NKp46 PE/Cy7 (#331915, BioLegend, San Diego, CA); NKp30 PE (#325207, BioLegend); NKp44 PE (#IM3710, Beckman Coulter, Brea, CA); CD94 PE (#130-098-974, Miltenyi Biotec, Auburn, CA); NKG2D APC (#120-003-706, Miltenyi Biotec); CD161 APC-Alexa Fluor750 (#B30630, Beckman Coulter); CD158e1 BV421 (#312713, BioLegend); CD158a,h PE/Cy7 (#A66899, Beckman Coulter); CD244 PerCp Cy5.5 (#B21171, Beckman Coulter); CD16 ECD (#A33098, Beckman Coulter); CD133 APC (#130-098-829, Miltenyi Biotec); CD57 BV421 (#563896, BD Biosciences); CD158 FITC (#339503, BioLegend).

### 2.5 Extracellular vesicle generation and isolation

NK3.3 and NK3.3-LTV cells (5 x 10^6^ cells/mL) were cultured in RPMI-1640 medium with 3% exosome-free FBS and 125 U/mL IL-2 overnight. The following morning, cell cultures were treated with phorbol 12-myristate 13-acetate and ionomycin for 5 h. HEK293 cells were cultured to ~80% confluency in DMEM medium with 3% exosome-free FBS for 48 h. Following incubation, all cell cultures were centrifuged at 500 g x 5 min, followed by 2,000 g x 10 min to remove cells and large cell debris. Supernatant was removed and filtered through a 0.22-µm filtration unit. EVs were precipitated using a polyethylene glycol (PEG)-acetate solution. A working solution of PEG was prepared using PEG8000 (MilliporeSigma) at 50% (w/v) with 0.4 M sodium acetate (MilliporeSigma), pH 8.5. For every 1 mL of EV supernatant, 200 µL of PEG-acetate (final concentration 10% (w/v) PEG, 0.08 M sodium acetate) was added and pipetted gently to mix. Once the PEG-acetate was completely incorporated, supernatants were refrigerated 24 h at 4°C. The next day, supernatants were centrifuged at 3,000 g x 10 min to pellet vesicles. 75% of supernatant was removed and remaining supernatant was used to wash sides of the tube. Supernatant was centrifuged once more at 3,000 g x 10 min. Any remaining precipitation solution was aspirated off carefully without disturbing the pellet. EV pellet was resuspended with PBS (10-15 µL PBS per 1 mL of supernatant). Protein content was measured using Pierce™ bicinchoninic acid (BCA) protein assay (Thermo Fisher Scientific). Bovine serum albumin was used as the standard.

### 2.6 Nanoparticle tracking analysis

EVs diluted in PBS were analyzed using the NanoSight NS300 analyzer (Malvern Panalytical, Malvern, United Kingdom) to determine size distribution as previously described (10). Briefly, 1 µg/µL EV protein was diluted 1000-fold in PBS. Samples were introduced using an automated syringe pump set to 40, camera level to 13, and detection threshold set to 3. Acquisition time was 60 s and at least 200 tracks were completed. Data analyzed using NTA 3.3 analytical software (Malvern Panalytical).

### 2.7 Immunoblot analysis

Cell and EV lysates (30 µg) were run on 12% SDS-polyacrylamide gels, then transferred to polyvinylidene difluoride (PVDF) membranes. Membranes were blocked with 1% bovine serum albumin blocking buffer for 30 min, then incubated with primary antibodies overnight at 4°C. After overnight incubation, membranes were washed three times, 10 min each, with 0.1% Tris-buffered saline with Tween 20 (TBST), followed by 1 h incubation with horseradish peroxidase (HRP)-conjugated secondary antibodies at room temperature. Membranes were washed an additional three times, 10 min each, in TBST and protein bands detected using Clarity™ Western ECL substrate (Bio-Rad, Hercules, CA). Blots were imaged on Chemidoc imaging system (Bio-Rad). The following primary antibodies were used for this study: Alix (#92880), CD63 (#52090), CD81 (#56039), GM130 (#12480), granzyme A (#4928), granzyme B (#4275), Perforin (#62550), and TSG101 (#72312, Cell Signaling Technology, Danvers, MA); DNAM-1 (#sc-376736, Santa Cruz Biotechnology, Dallas TX); NKLAM (made in-house); and β-actin (MilliporeSigma). The following secondary antibodies were used for this study: HRP-conjugated goat anti-mouse IgG and HRP-conjugated goat anti-rabbit IgG (#7076 and #7074, Cell Signaling Technology).

### 2.8 Calcein AM release cytotoxicity assays

K562, 8226/Luc, and U266 cells were co-cultured with NK3.3 and NK3.3-LTV cells to test cytotoxicity of NK3.3-LTV cells against tumor cells. All cells were placed into fresh media 24 h prior to assay setup. Tumor (target) cells were washed with R-2 media (RPMI-1640 medium without phenol red + 2% FBS) and resuspended in R-10 media (RPMI-1640 medium without phenol red + 10% FBS) with 5 µM calcein AM (Thermo Fisher Scientific). The cells were incubated for 40 min at 37°C in a humidified atmosphere of 5% CO_2_. After incubation, tumor cells were washed in R-2 media, resuspended in R-10 media, and viable cells counted in a hemocytometer using trypan blue dye exclusion. Tumor cells were plated 6,000 cells/well in 96-well clear, V-bottom microplates at various effector to target (E:T) cell ratios (20:1, 10:1, 5:1, 2.5:1). NK3.3 and NK3.3-LTV (effector) cells were washed with R-2 media and resuspended in R-10 media. Controls included a media control (100 µL target supernatant + 100 µL R-10 media (MC)); a maximum release control (100 µL target cells + 100 µL 5% Triton X-100 detergent solution (TX)); and a spontaneous release control (100 µL target cells + 100 µL R-10 media (SR)).

All assay conditions were set up in triplicate and repeated a minimum of three times. Microplates were incubated for 4 h at 37°C. After incubation, 100 µL of supernatant was collected from the top of the wells and transferred to 96-well black, flat-bottom microplates. The amount of calcein AM released from the target cells was detected using a BioTek Gen5 multi-detector microplate reader (Bio Tek, Winooski, VT). Percent specific lysis was calculated using the following formula: % specific lysis = [(mean of triplicates – mean of SR)/(max (mean of TX – mean of MC) – min (mean of SR – mean of MC))] x 100.

### 2.9 Cell viability and proliferation assays

WST-1 cell proliferation colorimetric assay was used to quantify proliferation, growth, and viability of K562, 8226/Luc, 8226/LR5, and 8226/B25 tumor cells treated with EVs. Cells were placed into fresh media 24 h prior to assay. 3,000 cells/well were seeded into 96-well clear, flat-bottom plates at final volume of 100 µL culture media. Cells were treated with PBS, HEK293 EVs (293 EVs), NK3.3 EVs (3.3 EVs) or NK3.3-LTV EVs (LTV EVs) at a low dose (25 µg/mL) and a high dose (100 µg/mL) over 72 h. 8226/Luc were also treated with bortezomib (25 nM) as a positive control. PBS was used as a negative control and 293 EVs served as a non-cytotoxic EV treatment. All treatment volumes were 10% of total culture volume. At the desired time point, 10 µL of WST-1 reagent was added to wells and incubated for 2 h. Absorbance was measured at 450 nm using a BioTek Gen5 microplate reader. All assay conditions were performed in triplicate and repeated a minimum of three times.

The viability and proliferation of U266, ARD, ARP, and MER MM cell lines were assessed by trypan blue dye exclusion. Cells were placed into fresh media 24 h prior to assay. 15,000 U266 cells/well were seeded into 96-well clear, flat-bottom plates at a final volume of 100 µL culture media. U266 cells were treated with either PBS, 293 EVs, LTV EVs, or bortezomib (25 nM) over 72 h. EVs were dosed at 25 µg/mL and 100 µg/mL. 6,000 ARD, ARP, and MER cells/well were plated into 96-well clear, flat-bottom plates at a final volume of 100 µL culture media and treated with PBS or LTV EVs (60 μg/mL) for up to 96 h. All treatment volumes were 10% of total culture volume. All assay conditions were set up in triplicate and repeated a minimum of three times.

The viability of CD38^+^ MM bone marrow cells from MM patients was also assessed by trypan blue dye exclusion. Variable numbers of primary MM patient cells were seeded into 96-well clear, flat-bottom plates at a final volume of 100 µL culture media (RPMI-1640 + 10% FBS) and treated with PBS or LTV EVs at 70 µg/mL over 72 h. Treatment volumes were 10% of total culture volume. Each day, cells were photographed, counted, and viability assessed by trypan blue dye exclusion. All assay conditions were set up in triplicate and repeated a minimum of three times.

Crystal violet assay was used to quantify proliferation, growth, and viability of hFF cells treated with EVs. 3,000 cells/well were seeded at a final volume of 100 µL in fresh media into 96-well clear, flat-bottom plates 24 h prior to addition of EV treatment. hFF cells were treated with PBS, 293 EVs, and LTV EVs at a low dose (25 µg/mL) and a high dose (100 µg/mL) over 72 h. PBS was used as a negative control, 293 EVs were used as a non-cytotoxic EV treatment. All treatment volumes were 10% of total culture volume. At the desired time point, media was removed from the wells and washed with PBS. Crystal violet stain was added to all wells for 10 min and then removed, followed by the addition of lysis buffer for 15 min. Absorbance was measured at 562 nm using a BioTek Gen5 microplate reader. All assay conditions were performed in triplicate and repeated a minimum of three times.

### 2.10 Caspase assays

Caspase-Glo 3/7 assay system was used to measure caspase-3 and -7 activity in RPMI-8226 cells (Promega, Madison, WI). 30,000 RPMI-8226 cells were seeded into 96-well clear, round-bottom plates at a final volume of 150 µL culture media. Cells were treated with PBS, 293 EVs (100 µg/mL), LTV EVs (100 µg/mL), or bortezomib (25 nM) over 72 h. All treatment volumes were 10% of total culture volume. At desired time point, cells were counted, centrifuged, and 5,000 cells/well were plated into 384-well, white plates. Caspase-Glo 3/7 reagent was added 1:1 to wells and incubated at room temperature for 1 h. Luminescence was measured using BioTek Gen5 microplate reader. Luminescent signal generated is proportional to the amount of caspase activity present. All assay conditions were performed in triplicate and repeated a minimum of three times.

### 2.11 Annexin V/7AAD staining

U266 cells were treated with PBS, 293 EVs (100 µg/mL), LTV EVs (100 µg/mL) or staurosporine (2.5 µM) over 72 h and monitored for apoptosis/cell death using Annexin V (#640908, BioLegend) with 7AAD staining (#559925, BD Biosciences). The assay was performed following manufacturer’s protocol and cells analyzed using an LSRII flow cytometer (BD Biosciences). Data were evaluated using FlowJo software.

### 2.12 Statistical analysis

All data were analyzed using Microsoft Excel (Redmond, WA). A Student’s *t*-test was used for statistical analysis with a *p* value ≤ 0.05 considered statistically significant.

## 3 Results

### 3.1 Comparison of immortalized NK3.3-LTV with non-immortalized NK3.3 cells

**Figure 1:**
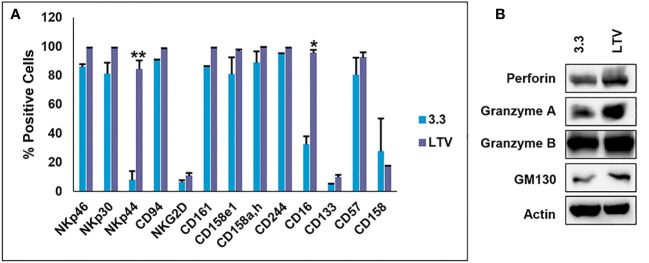
Characterization of the immortalized NK3.3-LTV cell line. **(A)** NK surface marker expression on non-immortalized (3.3) and immortalized (LTV) NK3.3 cells analyzed by flow cytometry. Data reported as percent positive cells; mean value ± SE (n=3, *p ≤ 0.001, **p ≤ 0.00001). **(B)** 3.3 and LTV whole cell lysate was examined by immunoblot analysis for NK cytotoxic proteins. Golgi membrane protein GM130 and actin were used as control markers. A representative blot is shown.

NK3.3, like other normal cell lines, undergoes growth arrest limiting cell expansion and EV production. To address this issue, we immortalized NK3.3 (LTV) with a lentivirus containing the catalytic subunit of human telomerase (*h*tert) to promote continual cell growth and expansion, providing an unlimited source for EV production. We compared the LTV cells to the non-immortalized NK3.3 (3.3) cells. **Figure 1A** shows percent positive cells by flow analysis of common NK surface markers. There was no difference between the LTV and 3.3 cells in cell surface expression of most of the NK markers examined. However, the expression of NK activating receptors, NKp44 and CD16, was significantly higher in LTV cells than in parental cells. **Figure 1B** shows expression of NK proteins associated with tumor killing (perforin and granzymes) in whole cell lysate from 3.3 and LTV cells. The Golgi membrane protein, GM130, was used as a control cell marker. Actin was used as a loading control. Immunoblot analysis showed no significant differences in protein expression between the two cell lines.

### 3.2 Anti-tumor killing activity of NK3.3-LTV cells

**Figure 2:**
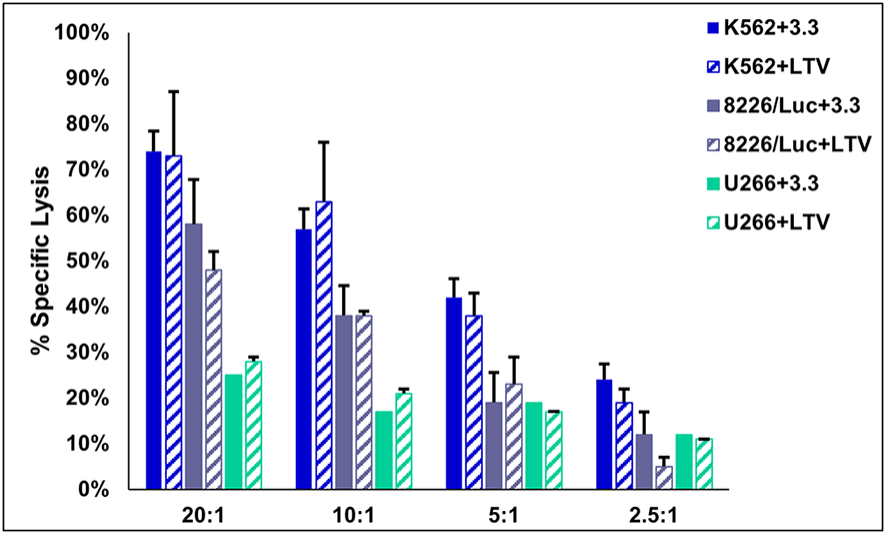
Cytotoxic activity of immortalized NK3.3-LTV cells. Effector (E) 3.3 and LTV cells were co-cultured with calcein AM-labeled tumor (T) cells for 4 h at various E:T ratios (20:1, 10:1, 5:1, 2.5:1). Calcein AM release was measured and percent specific lysis calculated. Data reported as mean value ± SE (n=3).

Based on the results in **Figure 1**, we sought to compare the cytotoxic activity of NK3.3-LTV cells and NK3.3 cells against several tumor cell lines. 3.3 and LTV cells were co-cultured with K562, 8226/Luc, and U266 cells at various E:T ratios beginning at 20:1. The cells were incubated for 4 h and cell lysis was measured. K562 was used as a control tumor cell line for cytotoxic activity because, as previously reported, NK3.3 cells actively target and induce cell death of K562 (31). There was no significant difference in percent specific lysis between 3.3 and LTV cells for all cell lines tested (**Figure 2**). LTV cells showed greatest cytotoxic activity against K562 (73% of cells killed), followed by 8226/Luc (48%), then U266 (28%) at the highest E:T ratio.

### 3.3 Immortalized NK3.3 EVs have a similar protein and particle profile as non-immortalized EVs

Previously, we prepared NK3.3 and 293 EVs by precipitation using a commercially available PEG polymer, ExoQuick-TC (System Bioscience, Palo Alto, CA) with a modified protocol. The isolated EVs were evaluated by transmission electron microscopy and nanoparticle tracking analysis (NTA), which identified a mixed population of membrane-bound spherical structures coinciding with the defined size ranges of both exosomes and microvesicles. The majority of EVs were exosomes, by both size and protein content (10). In this study, we isolated NK3.3, NK3.3-LTV and HEK293 EVs using our own PEG-acetate precipitation protocol.

**Figure 3:**
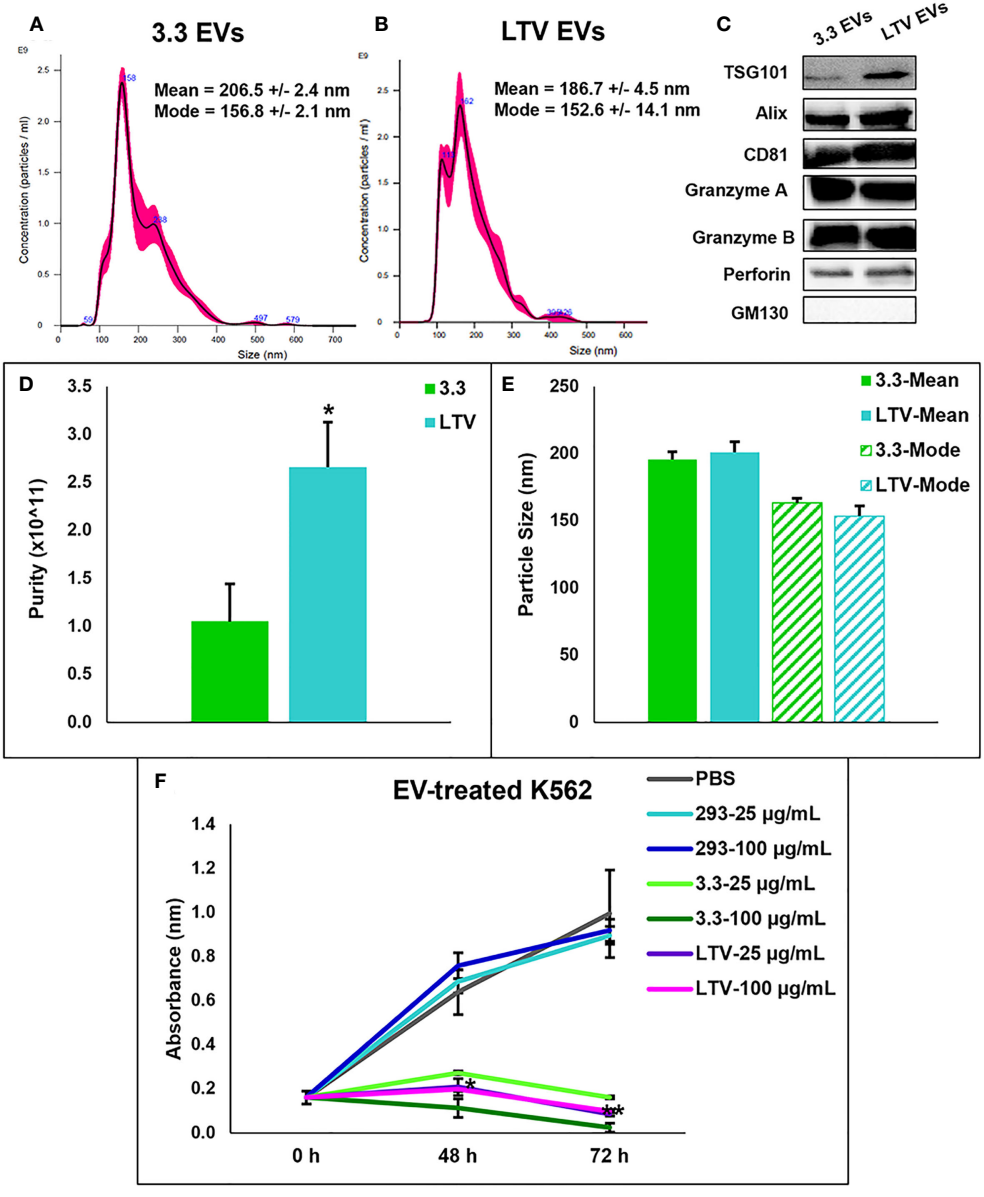
Characterization of NK3.3-LTV EVs isolated by PEG precipitation. NTA analysis of **(A)** 3.3 EVs and **(B)** LTV EVs isolated by PEG precipitation. Representative NTA plots are shown. **(C)** Immunoblot analysis of EV lysate (30 µg) comparing common EV- and NK-specific markers. GM130 was used as a negative EV control marker. A representative blot is shown. **(D)** EV purity was calculated as the ratio of particle concentration to protein (mean ± SE, n≥3, *p ≤ 0.05). **(E)** Average mean and mode particle size of 3.3 and LTV EVs. Data are representative of 3 or more measurements each. **(F)** K562 metabolic activity measured by WST-1 cell viability assay. Cells were seeded in 96-well plates and treated with either PBS, HEK293 EVs (293), NK3.3 EVs (3.3), or LTV EVs (LTV) at 25 µg/mL and 100 µg/mL over 72 h. PBS was used as a negative control. 293 EVs were used as a non-cytotoxic EV treatment. *p*-Values determined by comparison of LTV EV-treated cells to 293 EV-treated cells at each corresponding EV concentration and time point (mean ± SE, n=3, *p ≤ 0.0006, **p ≤ 0.000001).

NTA analysis depicted a similar size range of EVs derived from NK3.3 cells (**Figure 3A**) compared to EVs derived from NK3.3-LTV cells (**Figure 3B**). The majority of LTV EVs were 152 nm in size, represented by the highest peak, compared to 3.3 EVs at 156 nm.

Using a panel of EV- (TSG101, ALIX, CD81) and NK-specific (granzyme A, granzyme B, perforin) protein markers, immunoblot characterization of LTV EVs showed a similar protein expression profile compared to 3.3 EVs (**Figure 3C**). GM130, found in the Golgi matrix and not a component of EVs, was used as a marker of EV purity.

Another measure of EV purity is the ratio of particle concentration to protein (32). EVs isolated from LTV cells were significantly higher in purity than 3.3 EVs (**Figure 3D**). There was no significant difference in particle size between 3.3 and LTV EVs (**Figure 3E**).

We compared the cytotoxic effect of LTV EVs and 3.3 EVs on the tumor cell line, K562. Previously, we reported that NK3.3 EVs inhibited K562 cell growth and induced cytotoxicity *in vitro* (10). We treated K562 cells with 293, 3.3 or LTV EVs at a low dose (25 µg/mL) and a high dose (100 µg/mL) over 72 h. PBS was used as a negative control and 293 EVs were used as a non-cytotoxic EV treatment. Compared to 293 EV-treated cells, NK3.3 and NK3.3-LTV EVs killed K562 cells at both the low and high EV doses equally. (**Figure 3F**).

### 3.4 NK3.3-LTV EVs exhibit potent anti-tumor activity against MM cell lines and primary patient MM cells

**Figure 4:**
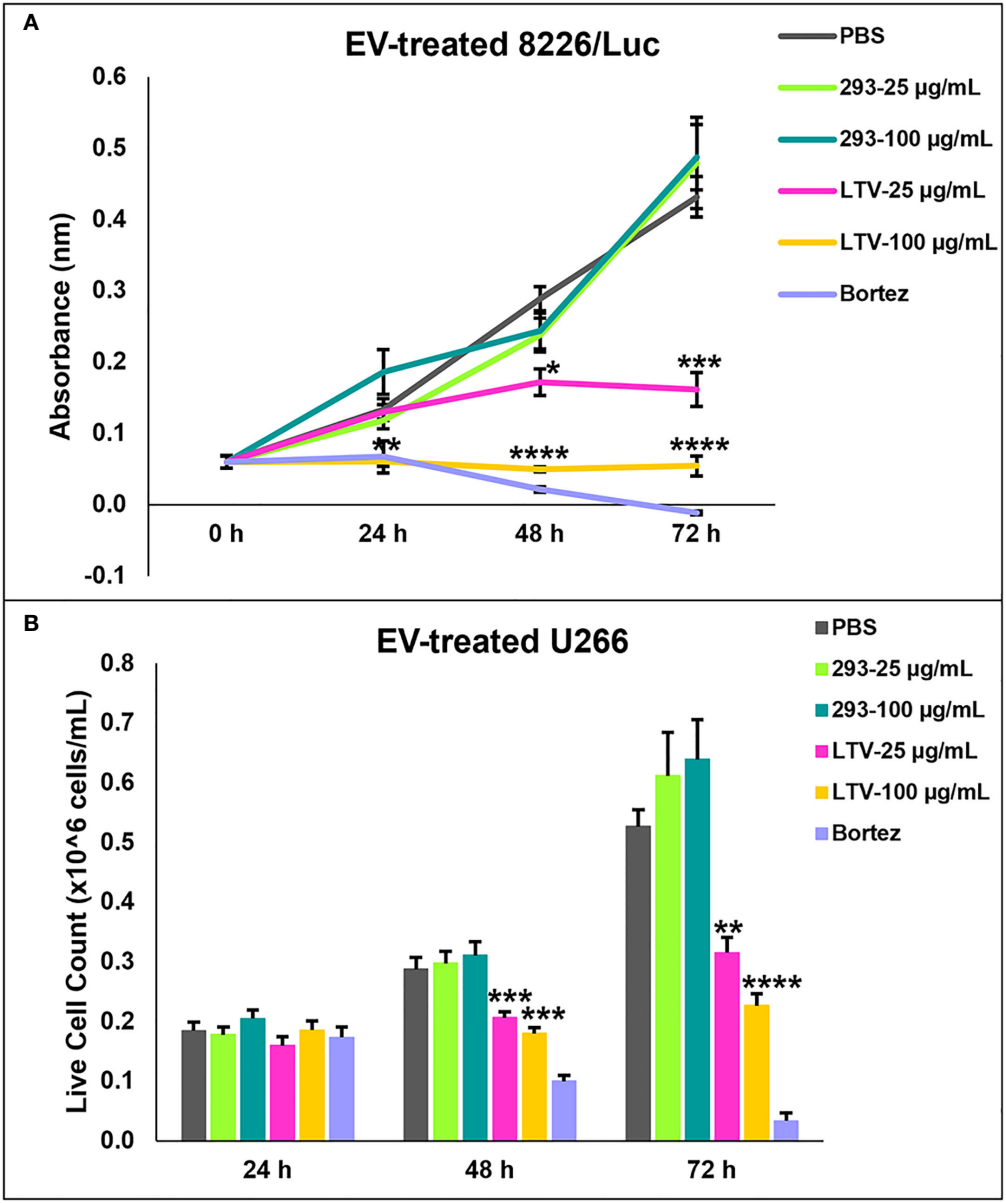
NK3.3-LTV EVs inhibit viability and proliferation of MM cell lines *in vitro*. 8226/Luc and U266 cells were seeded in 96-well plates and treated with either PBS, 293 EVs, LTV EVs, or 25 nM bortezomib (Bortez) over 72 h. EV doses were 25 µg/mL and 100 µg/mL. PBS was used as a negative control; 293 EVs were used as a non-cytotoxic EV treatment; bortezomib served as a positive control. **(A)** 8226/Luc metabolic activity was measured using WST-1 cell viability assay. **(B)** U266 live cell counts were determined by trypan blue dye exclusion. *p*-Values determined by comparison of LTV EV-treated cells to 293 EV-treated cells at each corresponding EV concentration and time point (mean ± SE, n=3, *p ≤ 0.04, **p ≤ 0.004, ***p ≤ 0.0005, ****p ≤ 0.00006).

We previously demonstrated that NK3.3 EVs exhibit a potent dose-dependent cytotoxic activity against multiple tumor cell lines, including K562, Jurkat (T cell leukemia), and breast cancer cell lines, MDA-MB-231 and MCF7 (10). Having established that NK3.3-LTV cells have similar anti-tumor cytotoxicity as non-immortalized cells and the EVs derived from them have similar protein expression, particle profile and killing of K562 as non-immortalized EVs, we sought to assess the anti-tumor efficacy of LTV EVs against MM. MM cell lines 8226/Luc and U266 cells were treated with PBS, 293 EVS, LTV EVs, or the proteasome inhibitor, bortezomib (25 nM) over 72 h. Cells were dosed with two EV concentrations, a low dose (25 µg/mL) and a high dose (100 µg/mL). The reduction in proliferation and/or cell viability induced by LTV EVs compared to 293 EVs was statistically significant beginning at 48 h (**Figure 4**). The corresponding 293 EV doses did not affect proliferation or cell viability. For both 8226/Luc and U266 cells, low dose LTV EVs decreased proliferation. The high dose of LTV EVs was cytotoxic to 8226/Luc and decreased proliferation of U266 cells. Bortezomib was cytotoxic to both cell lines. Representative images of LTV-EV treated MM cells are shown in **Supplementary Figure 1**.

**Figure 5:**
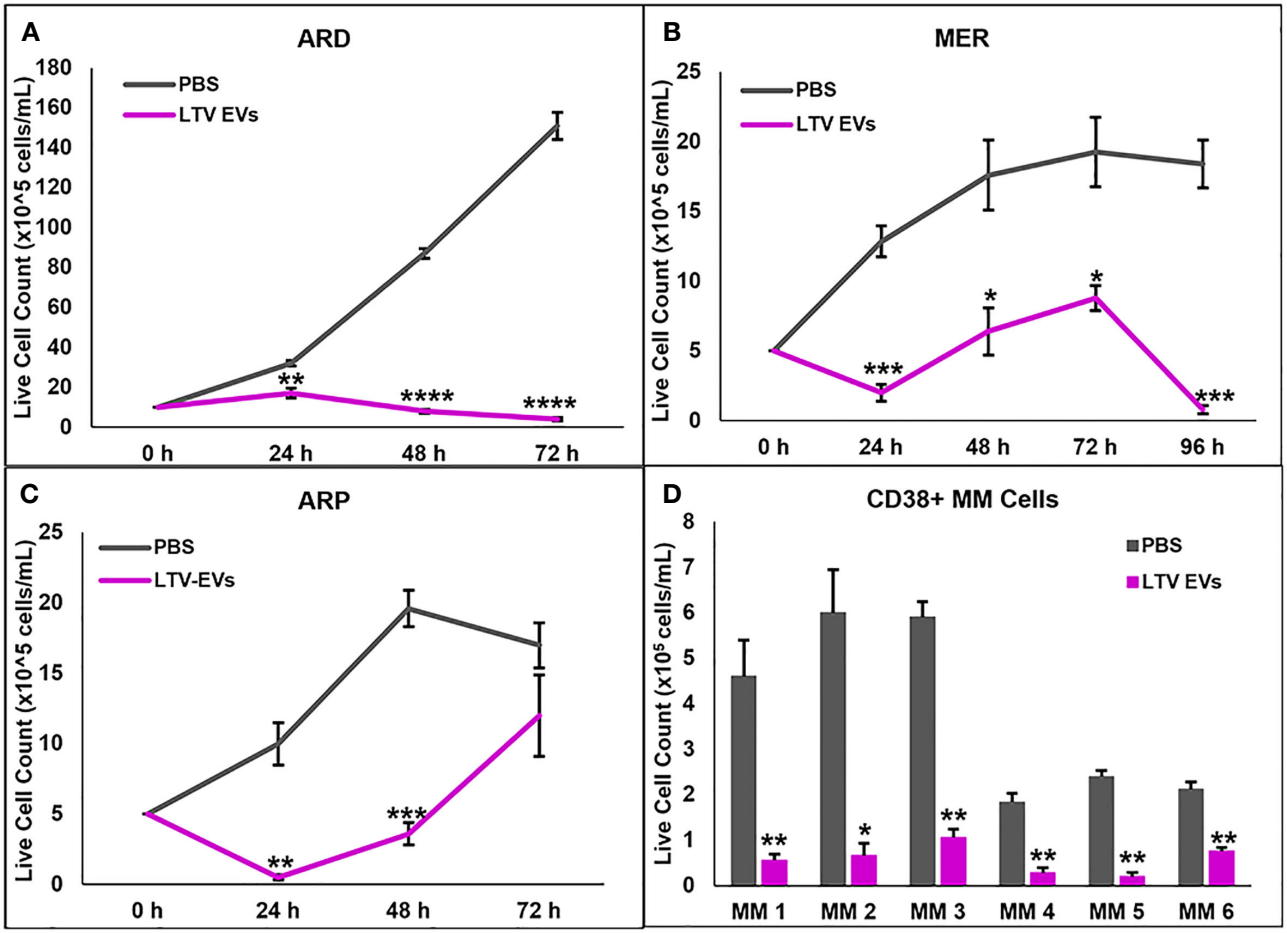
NK3.3-LTV EVs inhibit growth or induce death of patient MM cell lines and purified CD38^+^ MM cells from the bone marrow of patients with active disease. **(A–C)** ARD, MER, and ARP cells were seeded in 96-well plates and treated with either PBS or LTV EVs (60 μg/mL). Cell counts were performed every 24 h up to 96 h. *p*-Values determined by comparing LTV EVs to PBS-treated cells at the corresponding time point (mean cell counts ± SE, n=3, *p ≤ 0.01, **p ≤ 0.001, ***p ≤ 0.0001, ****p ≤ 0.00001). **(D)** Variable numbers of CD38^+^ primary patient MM cells were treated with PBS or LTV EVs (70 μg/mL). Cell counts were performed every 24 h up to 72 h. *p*-Values determined by comparing LTV EV to PBS-treated cell counts at 72 h (mean cell counts ± SE, n=3, *p ≤ 0.01, **p ≤ 0.001).

We also tested LTV EVs against cell lines we previously derived from MM patients: ARD, MER, and ARP. LTV EVs had significant anti-proliferative and/or cytotoxic activity against these MM cell lines, with some lines more sensitive than others (**Figures 5A–C**).

We next evaluated the ability of NK3.3-LTV EVs to kill primary patient MM cells, using CD38^+^ MM cells sorted from the bone marrow of patients with active disease. Primary MM cells from most patients survive for several days in culture but do not proliferate (33). MM cells from six of nine patient samples cultured remained viable over 3 days in control-treated wells. All six MM patient cells were significantly killed by NK3.3-LTV EV treatment (**Figure 5D**).

### 3.5 NK3.3-LTV EVs induce apoptosis in MM tumor cells

**Figure 6:**
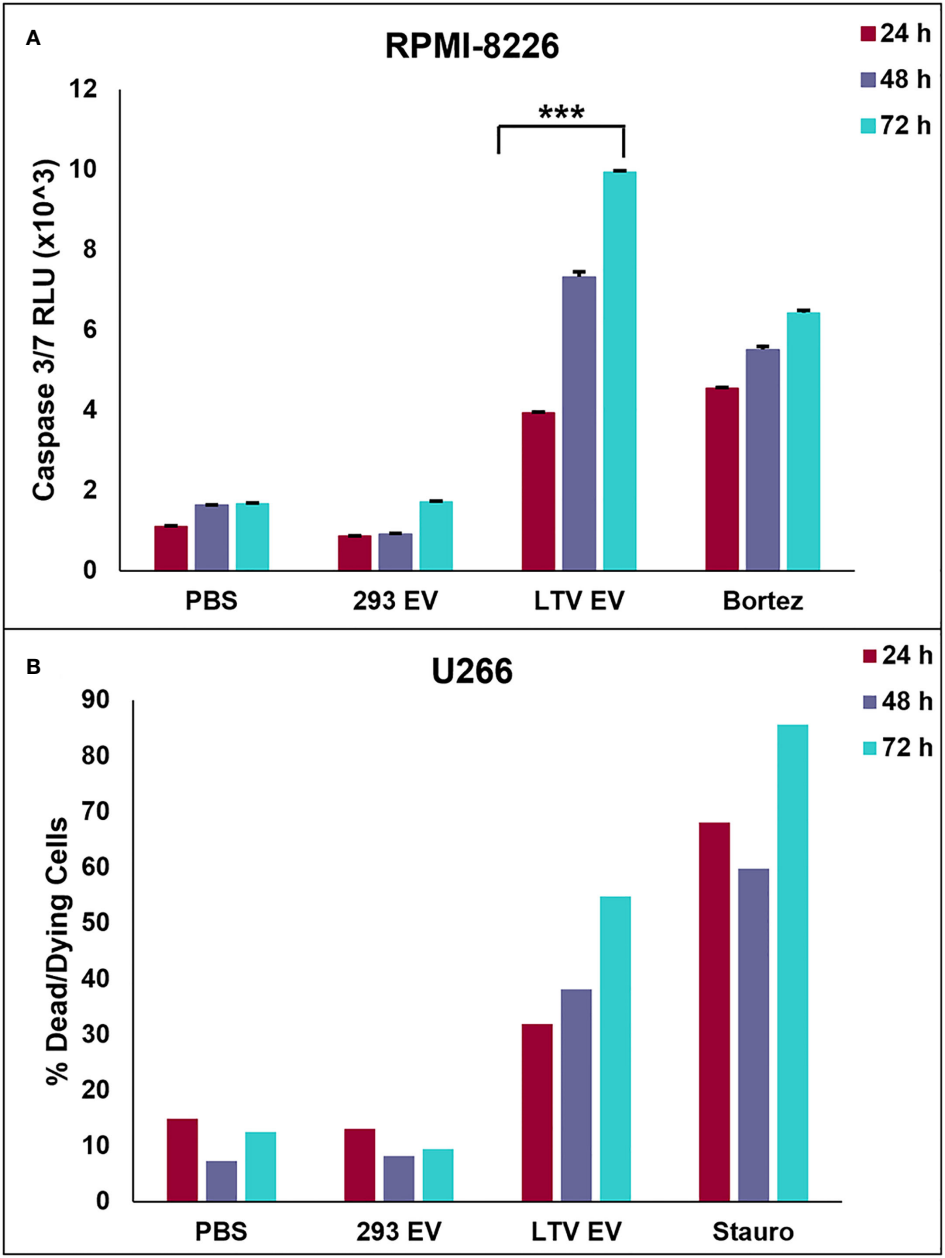
NK3.3-LTV EVs induce apoptosis in MM cells *in vitro*. **(A)** RPMI-8226 cells were treated with PBS, 293 EVs (100 µg/mL), LTV EVs (100 µg/mL), or bortezomib (25 nM) for 24, 48 and 72 (h) A DEVD pro-luciferin substrate was added to the cells at each time point; resulting luminescence was proportional to the amount of caspase 3/7 activity present. RLU: relative luminescence unit. (mean ± SE, n=3, ***p ≤ 0.0005). **(B)** U266 cells were treated with PBS, 293 EVs (100 µg/mL), LTV EVs (100 µg/mL), or staurosporine (2.5 µM) for 24, 48 and 72 (h) Cells were stained with annexin V and 7AAD at each tme point and analyzed by flow cytometry. Dead/dying cell frequency is the sum of all annexin V- and 7AAD-positive quadrants.

We next investigated whether LTV EVs induce death of MM cells by apoptosis. We used the luminescent caspase-glo 3/7 assay to measure caspase-3 and -7 activity in RPMI-8226 cells treated with LTV EVs. RPMI-8226 cells were treated with PBS, 293 EVs (100 µg/mL), LTV EVs (100 µg/mL), or bortezomib (25 nM) over 72 h. LTV EVs induced significant caspase 3/7 activity in RPMI-8226 cells compared to the non-cytotoxic EV treatment (293 EVs) (**Figure 6A**). Caspase activity increased in LTV EV-treated RPMI-8226 cells over 72 h.

We also performed annexin V/7AAD apoptosis assays of U266 cells treated with LTV EVs (100 µg/mL). Cells were stained with fluorochrome-conjugated annexin V and vital dye, 7AAD and analyzed by flow cytometry over 72 h to identify cells undergoing early apoptosis and late apoptosis/necrosis. LTV EV-treated cells induced more cell death compared to 293 EV-treated cells (**Figure 6B**). By 72 h, 55% of U266 cells were dead or dying. These results demonstrated that LTV EVs are cytotoxic to U266 cells. Scatter plots from a representative experiment show the frequency of live cells in the lower left quadrants, early apoptosis in the lower right quadrants, and late apoptosis/necrosis in the upper quadrants (**Supplementary Figure 2**).

### 3.6 NK3.3-LTV EVs inhibit proliferation and induce cytotoxicity in drug-resistant MM cells

**Figure 7:**
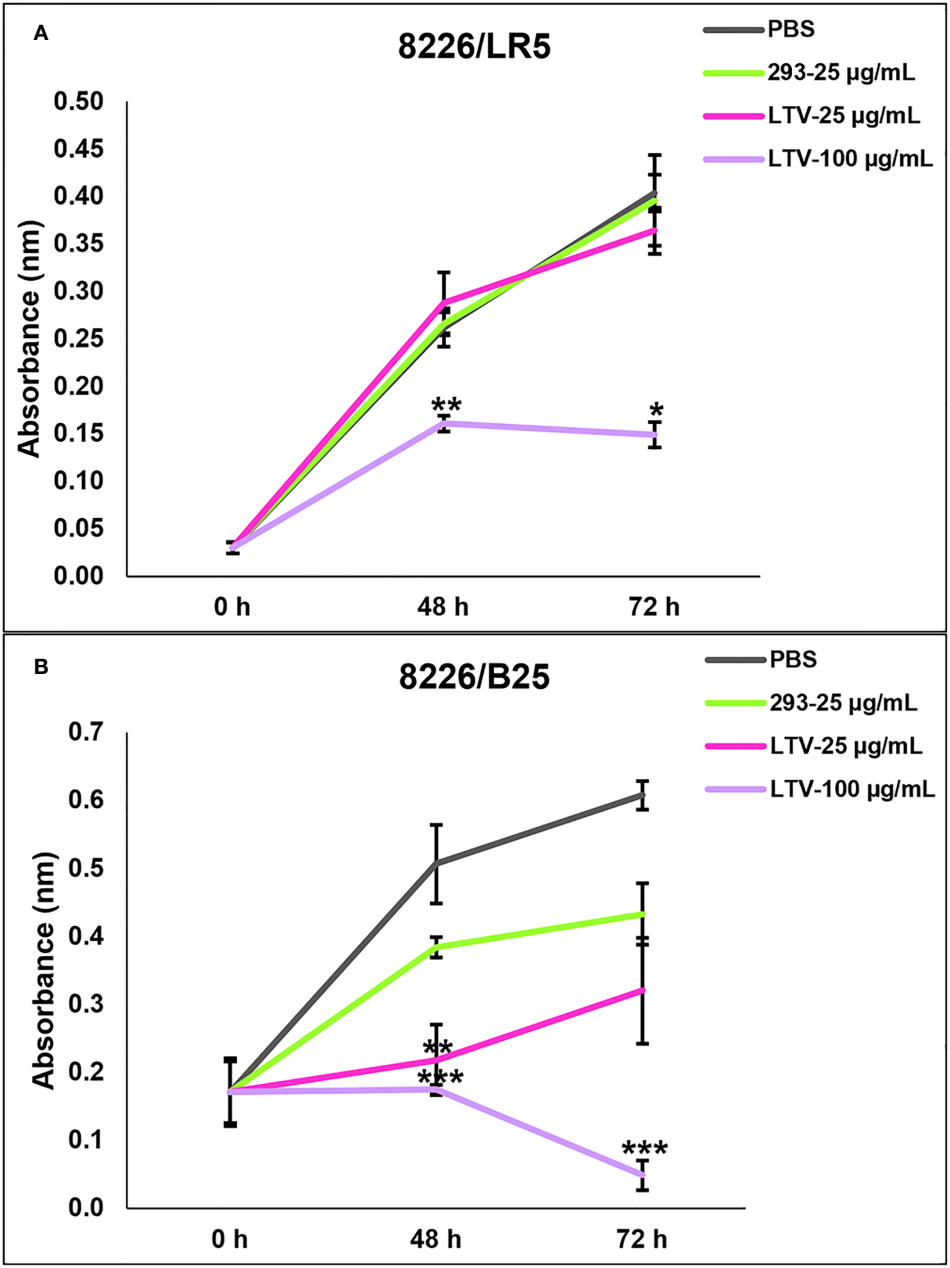
NK3.3-LTV EVs reduce viability and proliferation of drug-resistant MM cells *in vitro*. **(A)** Melphalan-resistant 8226/LR5 and **(B)** bortezomib-resistant 8226/B25 cells were seeded in 96-well plates and treated with PBS, 293 EVs or LTV EVs at 25 µg/mL and 100 µg/mL over 72 (h) Metabolic activity was measured by WST-1 cell viability assay. PBS was used as a negative control; 293 EVs were used as a non-cytotoxic EV treatment. *p*-Values determined by comparison of LTV EV-treated cells to 293 EV-treated cells at each time point (mean ± SE, n=3, *p ≤ 0.04, **p ≤ 0.004, ***p ≤ 0.0001).

Despite advancements in treatment modalities for MM patients, many ultimately develop multi-drug resistance, leading to triple-class refractory MM. These patients relapse, have a median survival rate of 5.6 months and desperately need new therapy options (5). Therefore, we evaluated the ability of NK3.3-LTV EVs to inhibit proliferation or kill drug-resistant MM cells. We used two drug-resistant MM cell lines, 8226/LR5 (melphalan-resistant) and 8226/B25 (bortezomib-resistant). Both cell lines were treated with LTV EVs at two concentrations over 72 h. Compared to the parental 8226 cell line (**Figure 4A**), the proliferation of melphalan-resistant 8226/LR5 cells was significantly inhibited only at the high dose of LTV EVs (**Figure 7A**). However, bortezomib-resistant 8226/B25 cells, like the parent culture, were very sensitive to LTV EVs. Low dose LTV EV treatment decreased proliferation; high dose LTV EV treatment was highly cytotoxic (**Figure 7B**). 293 EV treatment slightly reduced the proliferation of 8226/B25 cells compared to PBS-treated cells but this effect was not significant.

### 3.7 NK3.3-LTV EVs do not affect the proliferation/survival of normal cells

**Figure 8:**
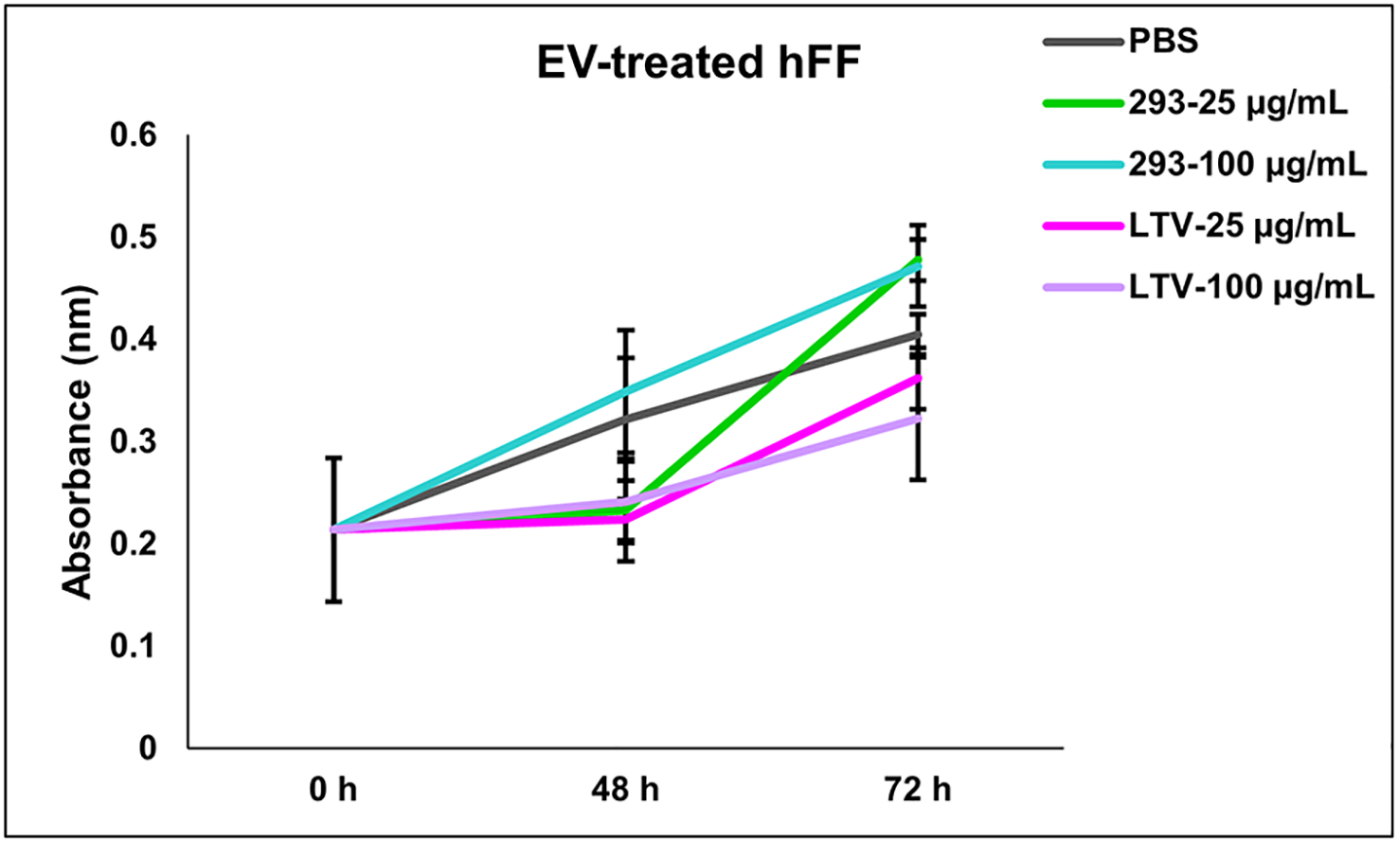
NK3.3-LTV EVs do not affect cell viability or proliferation of normal human foreskin fibroblast cells *in vitro*. hFF cells were seeded in 96-well plates and treated with either PBS, 293 EVs, or LTV EVs at 25 µg/mL or 100 µg/mL over 72 h. PBS was used as a negative control; 293 EVs were used as a non-cytotoxic EV treatment. Metabolic activity was measured by crystal violet staining (mean absorbance ± SE, n=3).

Previously, we tested the potential toxicity of NK3.3 EVs against three non-tumorigenic cell types: HEK293, activated peripheral blood lymphocytes, and cord blood lymphocytes. NK3.3 EV treatment had no effect on the metabolic activity or viability of these cells (10). Here, we expanded our analysis to test the potential toxicity of immortalized NK3.3 EVs against normal human foreskin fibroblast cells (hFF). hFF cells were treated with PBS, 293 EVs, or LTV EVs at two concentrations, low and high dose, over 72 h. There was no significant change in the viability or growth of LTV EV-treated hFF cells compared to control-treated cells, indicating a lack of toxicity (**Figure 8**).

## 4 Discussion

MM is a highly aggressive plasma cell neoplasm and, despite recent therapeutic advancements, remains incurable. Patients often relapse due to the development of multidrug resistance. Standard treatment for MM includes autologous stem cell transplantation, followed by high-dose chemotherapy in combination with immunomodulatory drugs and/or proteasome inhibitors. These drugs not only damage proliferating cancer cells, but also destroy normal dividing cells, causing severe decline in patients’ health. Because of adverse side-effects, there is a need to develop new treatments that are highly specific towards cancerous cells without harming healthy cells (34).

A promising new cancer immunotherapeutic is the use of EVs. EVs are nano-sized membrane-bound vesicles that contain cargo from the cell which they originate. Upon interaction with target cells, EVs can mediate therapeutic effects through various mechanisms (35). Since EVs occur naturally in the bloodstream and tissues, administration of EVs is thought to be safe and elicit few toxic or inflammatory effects, making them a promising new cell-free therapeutic (36). To date, most clinical studies use EVs derived from mesenchymal stromal cells. These studies have shown beneficial results without adverse side effects (19).

EVs derived from NK cells express typical exosomal markers (TSG101, ALIX, CD63) as well as NK cell receptors (NKp30, NKp44, NKp46, NKG2D, and DNAM1) (7, 37, 38). Studies have shown that NK EVs contain perforin, granzyme A, granzyme B, granulysin, and FasL, proteins that activate both intrinsic and extrinsic apoptosis pathways in cancer cells (7, 37, 38). NK EVs exhibit a dose- and time-dependent cytotoxic effect *in vitro* against a variety of cancer cell lines: breast (11–16), glioblastoma (11, 12, 16), leukemia (15, 17, 18), colon (11, 12), ovarian (11, 14), liver (12, 39), melanoma (11), prostate (11), stomach (12), thyroid (16), cervical (18), and lung (18). Importantly, NK EVs do not kill normal, healthy cells (10, 17).

NK EVs, for targeted cancer therapy, are often isolated from peripheral blood mononuclear cells (PBMCs) or NK92 cells. NK cells isolated from cancer patients may be dysfunctional, and donor NK cells exhibit donor-dependent functionality (20, 37). Due to cellular source and physiological conditions, NK EVs are extremely heterogeneous in contents, size, and membrane composition, resulting in variability across studies and reduction in reproducibility (19, 20, 35, 37). PBMCs require timely and expensive activation/expansion protocols to obtain enough cytotoxic NK cells to generate EVs for treatment. NK92 is an Epstein-Barr virus positive lymphoma cell line; many tumor cells have been shown to release EVs that promote tumor growth and spread (40, 41).

This is the first study to examine NK EVs derived from an immortalized non-tumorigenic NK cell line to target and kill MM. The use of an immortalized cell line to produce NK EVs reduces NK donor heterogeneity, eliminates lot-to-lot product variability, and decreases costs and ease of manufacturing EVs (19). We immortalized the human NK cell line, NK3.3, to overcome cell exhaustion and promote continual cell expansion. NK3.3 is the only non-tumorigenic NK cell line of its kind. Originally isolated from the peripheral blood of a healthy donor, immortalization has maintained high levels of cell growth with no change in function for over 8 months (**Figures 1**, **2**). We are continuously monitoring NK3.3-LTV cell growth and assessing cell function, surface marker expression, and protein expression every 6 months.

We previously reported an extensive analysis of EVs generated from NK3.3 cells (10). We compared NK3.3 EVs to NK92- and peripheral blood NK cell-derived EVs and showed strong similarities in protein profile and cytotoxic activity. NK3.3 EVs contain common EV proteins (CD63, TSG101, HSP70, and ALIX) as well as important cytotoxic proteins: perforin, granzyme A, granzyme B, and granulysin. NTA analyses of NK3.3, NK92, and peripheral blood NK cell derived EVs revealed a similar size distribution of EV particles (10). Proteomic analysis of NK3.3 EVs identified high expression of adhesion molecules, ICAM1 and VCAM1, which may be important for EV uptake. DNAM1 is also highly expressed by NK3.3 and may be important in NK3.3 anti-tumor activity (10). RNA sequencing of NK3.3 EVs identified miRNAs expressed in NK cells that regulate NK function, granzyme B and perforin expression, and IFN-γ production. NK3.3 EVs also contain miRNAs with tumor suppressor activity, which may contribute to the specificity of NK EVs for tumor killing (10). NK3.3 EVs induce caspase 3/7 activity in K562 (leukemia), Jurkat (T-cell leukemia), MDA-MB-231 (breast cancer) and MCF7 (breast cancer) (10).

Comparison of immortalized NK3.3 EVs (LTV EVs) to non-immortalized NK3.3 EVs (3.3 EVs) showed similarities in protein expression, particle concentration and size, and anti-tumor activity (**Figure 3**). LTV EVs were highly effective in decreasing cell proliferation and inducing cytotoxicity in a variety of MM cell lines, including primary patient MM cells (**Figures 4**, **5**). LTV EVs also induced caspase-3 and -7 activity in RPMI-8226 cells (**Figure 6**). Compared to non-immortalized 3.3 EVs, LTV EVs contain higher concentrations of perforin and granzymes A and B. Perforin forms pores in the tumor cell membrane, allowing granzymes to enter and induce caspase-dependent and -independent apoptosis (7). NK EVs with higher levels of perforin and granzyme B have been shown to have more potent anti-tumor activity than EVs with lower amounts, suggesting that these proteins are critical mediators of cell death (14). In caspase-dependent apoptosis, granzyme B causes a signaling cascade disrupting mitochondria and releasing cytochrome c, activating caspase-3, -7, and -9 to induce apoptosis (7). Pan-caspase inhibitors partially, but significantly reduce NK EV-mediated tumor killing (16, 38). Our data indicate significant long-term caspase 3/7 activity in LTV EV-treated tumor cells. Of the multiple proteins and miRNAs packaged within NK EVs, it is likely that several, working alone or together, participate in the killing process. NK3.3 and its EVs do not express FasL. Wu et al. found that the protein level of FasL in NK EVs was negligible, and its level did not correlate with cytotoxicity. They similarly suggested that there is not a single mediator of tumor killing but that perforin, granzyme A, granzyme B and granulysin in NK EVs together play prominent roles in inducing cytotoxicity (38). Studies are in progress to further identify the role of various EV constituents in NK3.3 EV-mediated killing of MM.

Tumor cells interact with surrounding cells and tissues in the tumor microenvironment (TME) and release EVs with tumor-promoting activity. Tumor-derived EVs also suppress recruitment and function of immune effector cells. Together, the TME and tumor-derived EVs create an acidic, hypoxic environment that is inhibitory to NK cell activation and cytotoxic function (34). These factors contribute to tumor cell survival and drug-resistance. MM patients often develop resistance to first generation immunomodulatory drugs and proteasome inhibitors (2). Understanding the mechanism of resistance is important to developing new therapeutics to overcome tumor cell drug resistance and improve survival of triple-class refractory MM patients. In this study, we evaluated the ability of NK3.3-LTV EVs to kill two drug-resistant MM cell lines, 8226/LR5 (melphalan-resistant) and 8226/B25 (bortezomib-resistant). Melphalan is an alkylating chemotherapeutic agent that induces irreparable DNA damage. Bortezomib is a first-class proteasome inhibitor. It has a short peptide-based structure that binds to catalytic sites on proteasomes, causing excessive accumulation of proteins within MM cells, inducing ER stress and apoptosis (5). In **Figure 7A**, the high dose of LTV EVs significantly decreased proliferation of 8226/LR5; the low dose had no effect on cell proliferation or induction of cell death. In contrast, bortezomib-resistant 8226/B25 cells responded to LTV EV treatment like the parent 8226 culture; low dose LTV EVs decreased proliferation and the high dose was highly cytotoxic (**Figure 7B**). These results indicate differences between melphalan and bortezomib-resistant 8226 cells in their sensitivity to LTV EV killing. Similar results were seen with 8226/Luc and U266 cells (**Figure 4**). 8226/Luc cells were more sensitive to both NK cell- and NK EV-mediated killing than U266 cells. U266 cells may be more resistant to NK and NK EV killing mechanisms due to low expression of NK receptor ligands or by their ability to release tumor-derived EVs that block the signals that activate NK cells and NK EVs, diminishing cytotoxicity (34). The basis of the differences in sensitivity to NK EV-mediated killing is being explored. Studies are also being conducted to assess apoptosis and other pathways of cell death in drug-resistant and NK-resistant cell lines treated with NK EVs.

An important take-away from this study is that immortalized NK-derived EVs do not harm normal cells (**Figure 8**). It is unclear how NK EVs target and kill tumor cells but spare normal cells. Some studies reported that NK EVs can be internalized by healthy cells without inducing cytotoxicity (8, 16). Samara et al. showed selective EV uptake by cancer cells (17). It was reported that NK EV-mediated tumoricidal activity could be partially inhibited by blocking DNAM1 or NKG2D (11, 18). NK cells and their EVs express multiple receptors and adhesion molecules; some receptor-ligand interactions may be tumor or tumor cell specific. Studies are in progress to fully characterize the surface receptor(s) and cytotoxic molecules involved in NK EV-mediated killing of MM cells.

*In vivo* studies are currently ongoing to evaluate the ability of LTV EVs to delay/prevent tumor recurrence in a murine minimal residual disease model of human MM. We envision the use of NK EVs to treat patients in the setting of stable or minimal residual disease. There are challenges to the use of NK EVs which need to be addressed and overcome. Currently, there is no gold standard for the isolation and purification of NK EVs. It is unclear whether different isolation methods and culture protocols affect NK EV cargo and function. This makes it difficult to compare the benefits of NK EV therapy among studies. NK EVs have been studied extensively *in vitro* as a cancer therapeutic, but there are limited *in vivo* studies that evaluate efficacy, biodistribution, stability and toxicity (34, 35).

Overall, NK3.3-LTV EVs display strong anti-tumor activity against MM cell lines and primary patient cells, including drug-resistant MM cells. Tumors often develop mechanisms to escape killing and gain resistance to drug and immunotherapy, leaving patients with limited treatment options. The use of immortalized NK3.3 EVs may be a safe and effective new treatment for MM, eliminating the need to isolate cells from different donors, reducing heterogeneity, and decreasing lot-to-lot variability of EV products.

## Data availability statement

The raw data supporting the conclusions of this article will be made available by the authors, without undue reservation.

## Ethics statement

The studies involving humans were approved by University of Arkansas for Medical Sciences IRB. The studies were conducted in accordance with the local legislation and institutional requirements. The human samples used in this study were acquired from a by- product of routine care or industry. Written informed consent for participation was not required from the participants or the participants’ legal guardians/next of kin in accordance with the national legislation and institutional requirements.

## Author contributions

EM: Data curation, Formal Analysis, Investigation, Methodology, Validation, Writing – review & editing, Writing – original draft. JK: Data curation, Formal Analysis, Investigation, Methodology, Validation, Writing – review & editing, Conceptualization, Funding acquisition, Project administration, Resources, Supervision.

## Supplementary material

Supplementary Figure 1 Representative photographs of untreated and NK3.3 EV-treated **(A)** 8226/Luc, U266 and **(B)** primary MM cells at 72 h. Cells in 96 well plates were photographed using an inverted phase microscope. Higher magnification pictures of untreated and NK3.3 EV-treated primary MM cells illustrate the killing activity of NK3.3 EVs.

Supplementary Figure 2 Representative scatter plots of EV-treated U266 cells demonstrating LTV EV-induced apoptosis. U266 cells were treated with PBS, 293 EVs, LTV EVs, or staurosporine over 72 h, stained for annexin V/7AAD and analyzed by flow cytometry. Lower left quadrants = live cells; lower right quadrants = cells in early apoptosis; upper quadrants = cells in late apoptosis/necrosis.
