# Supplementary figures and images for: Extracellular vesicles derived from immortalized human natural killer cell line NK3.3 as a novel therapeutic for multiple myeloma
Source: Front Immunol. 2023 Sep 25;14:1265101. doi: 10.3389/fimmu.2023.1265101 (PMC10560732; doi:10.3389/fimmu.2023.1265101)

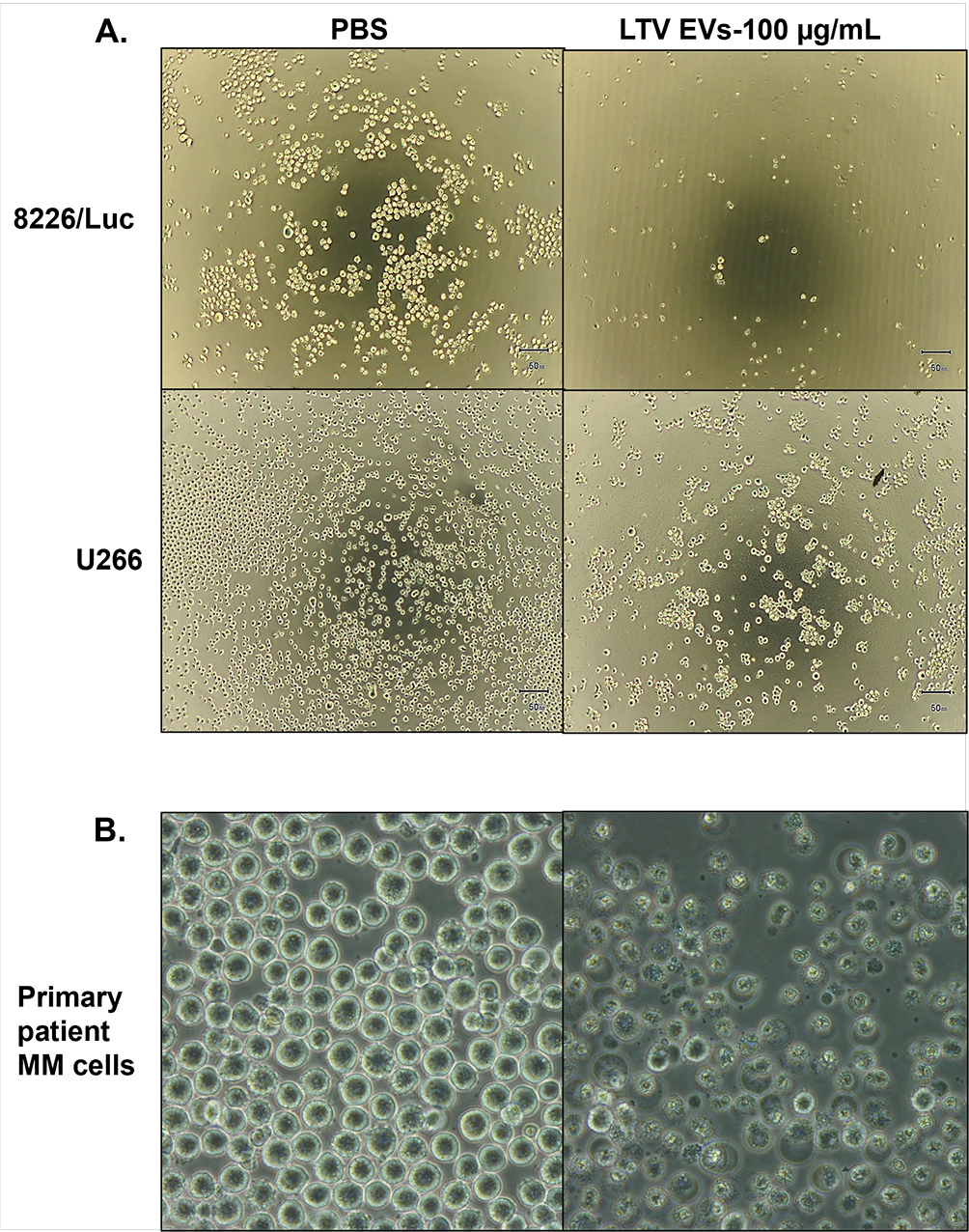

Supplement: Supplementary Figure 1 — Representative photographs of untreated and NK3.3 EV-treated (A) 8226/Luc, U266 and (B) primary MM cells at 72 h. Cells in 96 well plates were photographed using an inverted phase microscope. Higher magnification pictures of untreated and NK3.3 EV-treated primary MM cells illustrate the killing activity of NK3.3 EVs. [file Image_1.tif]

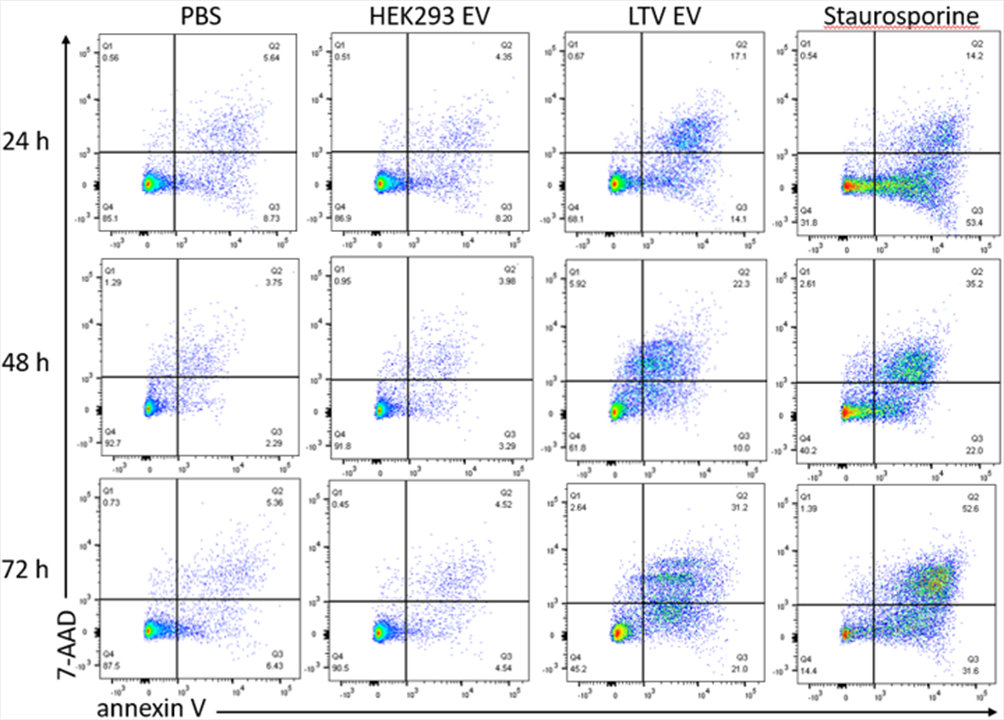

Supplement: Supplementary Figure 2 — Representative scatter plots of EV-treated U266 cells demonstrating LTV EV-induced apoptosis. U266 cells were treated with PBS, 293 EVs, LTV EVs, or staurosporine over 72 h, stained for annexin V/7AAD and analyzed by flow cytometry. Lower left quadrants = live cells; lower right quadrants = cells in early apoptosis; upper quadrants = cells in late apoptosis/necrosis. [file Image_2.tif]
